# Veterinarians’ perception of livestock infectious disease: results from a five country cross-European survey (2024)

**DOI:** 10.1186/s12917-026-05410-1

**Published:** 2026-03-28

**Authors:** Alistair Antonopoulos, Sharon Sweeney, Kelly McCall, Edgar Garcia Manzanilla, Carla Correia-Gomes, Alison Burrell, Erika Chenais, Lena-Mari Tamminen, László Ózsvári, Johannes Charlier, Stelian Bărăităreanu, Jeroen Dewulf, Evelien Biebaut, Áine Regan

**Affiliations:** 1 Kreavet BV, Kruibeke, Belgium; 2 Department of Agri-food Business & Spatial Analysis, Rural Economy and Development Programme, Teagasc, Mellows Campus, Athenry, Co. Galway H65 R718 Ireland; 3 Pig and Poultry Research and Knowledge Transfer Department, Teagasc, Moorepark, Fermoy, Co. Cork P61 C996 Ireland; 4 School of Veterinary Medicine, University College Dublin, Dublin 4, Dublin Ireland; 5 Animal Health Ireland, Carrick-on-Shannon, N41WN27 Ireland; 6 Department of Epidemiology, Disease Surveillance and Risk Assessment, SVA, Uppsala, 751 89 Sweden; 7 Department of Biosciences, SLU, Uppsala, 750 07 Sweden; 8 Department of Clinical Sciences, SLU, Uppsala, 750 07 Sweden; 9 Department of Veterinary Forensics and Economics, University of Veterinary Medicine Budapest, Istvan u. 2, Budapest, 1078 Hungary; 10 Faculty of Veterinary Medicine, University of Agronomic Sciences and Veterinary Medicine of Bucharest, Bucharest, Romania; 11 Department of Internal Medicine, Reproduction and Population Medicine, Faculty of Veterinary Medicine, Ghent University, Merelbeke, Belgium

**Keywords:** Stakeholder survey, Veterinary, Transboundary disease, Livestock disease, Infectious livestock disease

## Abstract

**Background:**

Livestock production accounts for nearly half of all agricultural output globally, but the long-term sustainability of the sector is threatened by a range of infectious diseases. Endemic diseases lead to a wide range of production losses and animal welfare issues, while transboundary and epidemic diseases can lead to widespread deaths, the implementation of stringent control measures, and a disruption of trade. Zoonotic diseases further threaten human health. It is therefore critical that livestock diseases are effectively controlled. When implementing disease control strategies and biosecurity measures, it is important to understand stakeholders’ views, particularly regarding which diseases should be prioritised.

**Methods:**

The current study aims to develop our understanding of veterinarians’ views regarding infectious diseases in livestock across Belgium, Ireland, Hungary, Romania, and Sweden through an online survey. The survey examines veterinarians’ perception of the risk posed by infectious livestock diseases based on a three-part score comprising of: the perceived likelihood of an outbreak occurring; the impact of an outbreak; and the ease of controlling an outbreak. Veterinarians’ awareness of given diseases was also assessed.

**Results:**

We report a high degree of variation in the perception of risk for livestock diseases across both the livestock production sectors examined, and countries. The transboundary diseases African swine fever, foot and mouth disease and avian influenza were seen as posing a high risk overall, however, bluetongue, lumpy skin disease, peste des petits ruminants, and sheep and goat pox showed a highly regional variation. Endemic diseases were often seen as posing a higher risk than transboundary diseases in many countries. We further identified several awareness gaps, particularly related to zoonotic diseases such as Hepatitis E, *Campylobacter* and *Salmonella* Dublin.

**Conclusions:**

Perception of risk for both transboundary and endemic diseases show a high degree of variation at the national level, often reflecting differences in the importance of production sectors by country. Critical awareness gaps for both transboundary, and zoonotic, diseases require further investigation.

**Supplementary Information:**

The online version contains supplementary material available at 10.1186/s12917-026-05410-1.

## Background

Livestock production worldwide accounts for almost half the total value of agriculture [1], with that figure being even higher in industrialised countries [2]. Infectious livestock diseases, however, threaten both commercialised intensive and smallholder production systems [3] and further raise concerns for the long-term sustainability of the sector as a whole [4]. Increasing competition for natural resources and the requirements to manage carbon emissions from the agricultural sector place further constraints on livestock production in Europe and beyond [2]. Apart from the direct impacts of animal infectious disease, such as production losses and animal welfare issues [3, 5], outbreaks might also cause trade disruptions [3, 6]. This is particularly tangible for outbreaks of transboundary animal infectious diseases such as African swine fever (ASF) foot and mouth disease (FMD) [6, 7] and peste des petits ruminants (PPR) [8]. Infectious livestock disease also contributes to increased use of antimicrobials, which is a driver of selection for resistance [9, 10]. This leads to an increase in the prevalence of resistant pathogens in animals and raises the risk of transmission of resistant genes and bacteria to humans and the environment.This, in turn necessitates stronger animal health measures, targeted stewardship, vaccination and diagnostics, and integrated surveillance to mitigate AMR [11]. Finally, animal infectious diseases can also pose a serious threat to human health (i.e., zoonotic diseases), with an estimated 60% of emerging human pathogens being zoonotic [12]. Spillover from wildlife or domesticated animals can lead to severe global pandemics, such as the H1N1 influenza A pandemic of 2010 and, most recently, the SARC-CoV-2 pandemic in the early 2020s [13]. This can be further compounded by the capacity for certain animal species to serve as bridging or amplifying hosts [14] or mixing vessels harbouring multiple strains of a virus, facilitating viral reassortment and the potential for increased virulence [15, 16]. Beyond global outbreaks, zoonotic disease can also lead to more localised national outbreaks, such as the Q-fever outbreak in the Netherlands in 2008 [17]. Additionally, there are a wide range of zoonotic diseases endemic to livestock which can cause severe disease and deaths in humans. These include *Campylobacter* [18], *Salmonella* [19], Hepatitis E [20], tuberculosis [21], and multiple *E. coli* serovars [22]. It is therefore of critical importance for both human and animal health that livestock diseases are effectively controlled. Control is, however, complex, with a wide range of available measures to combat a myriad of infectious diseases with greatly differing aetiologies and transmission routes. These measures can include surveillance, both for early detection of outbreaks and routine diagnostic monitoring; pharmaceuticals or vaccination; biosecurity; and, in the case of outbreaks of disease that are included in EU and national animal health laws and thus subject to specific regulations, culling and movement restrictions. Biosecurity aims to prevent the spread of pathogens into, within, and from an animal population [23, 24].

When designing disease control strategies, it is of importance to both understand which diseases should be prioritised, particularly in order to best direct often limited resources [25–28], and to understand stakeholders’ views related to which diseases pose the greatest risk to human and animal health or that are prioritised for other reasons [28, 29]. Stakeholder perspectives are of particular importance for understanding the implementation of biosecurity measures or vaccination programmes [30, 31]. This includes both clinical stakeholders such as veterinarians [30, 32–34] and livestock production stakeholders in general [30]. To date there have been a range of studies carried out concerning European farmers’ risk perception of livestock disease risks, their attitudes related to risk management and biosecurity, and the impact of farmers’ perceptions on the uptake of control measures. Many studies have focused on a specific sector, for example, pigs [35, 36], cattle and small ruminants [37, 38], or poultry [39, 40], and often only considered the situation within a single country [35–40]. However, in general, there has to date been less focus on disease risk perception amongst veterinarians. It has previously been reported that veterinarians often are one of the most trusted sources of information for farmers [41], and that they have an important role to play in communicating disease control and prevention messages to farmers [42, 43]. Thus, improved understanding of veterinarians’ perception of livestock infectious disease risks can serve to improve the uptake and implementation of farm biosecurity measures across Europe. There are studies which have considered veterinarians, farmers and associated stakeholders [30] or that have focused on veterinarians’ perception of the zoonotic infection risk to their own health [44], but as far as the authors are aware, there are no Europe-wide studies examining veterinarians’ risk perception of a range of priority diseases and across multiple livestock sectors. This European-wide survey of veterinarians’ perception of risk in connection to a range of epidemic, endemic, and zoonotic diseases of livestock builds on previous work establishing a list of prioritised diseases of importance to European livestock production [45] and has the objective to improve our understanding of veterinarians’ perception of the risk posed by livestock disease at the national level within a cross section of European countries.

## Methodology

### Study design, participants and data collection

The study reports on the data collected in the Horizon Europe BIOSECURE project (https://biosecure.eu/) from a cross-sectional survey administered to veterinarians working in the livestock sector in different European countries. For the purposes of this study, the livestock sectors considered were cattle, poultry, small ruminants, and pigs as they are the main terrestrial livestock sectors in Europe. This survey was part of a larger study exploring the attitudes and risk perceptions of farmers and veterinarians towards animal health diseases in different European countries [46]. The survey was designed initially in English and pre-tested in the Irish context with farmers and farm advisors (*n* = 8) and in the European context with veterinarian scientists and biosecurity experts (*n* = 5). It then underwent a process of back-translation [47] and local language piloting in each of the additional countries, facilitated through co-author leads in each country.

The survey was administered electronically between May and November of 2024, using convenience sampling through local veterinarian networks via a link by the local co-authors in each participating country: Belgium, Hungary, Ireland, Romania, Spain, Italy, and Sweden. Inclusion criteria included being a qualified veterinarian working in at least one of the four sectors under study in the participating countries. Informed consent was obtained from all participants prior to completion of the survey, as participants were unable to proceed to the full survey without first providing consent to participate. Participation was voluntary, and participants could withdraw at any time during the survey. For informed consent, survey introduction, and the full survey see Supplementary Materials 3.

### Survey distribution

In Belgium, the survey was distributed through two private companies: one operating in the cattle sector, which shared the survey with its veterinarians, and another specialising in animal health support, which distributed it via their newsletter. The survey link was further shared via social media posts, and personal contacts were approached to supplement data collection.

In Hungary, the survey was primarily distributed through two intermediaries: the alumni e-mail database of the University of Veterinary Medicine Budapest and two closed online forums for veterinarians, namely Hungarovet and Vetmail. These platforms distributed the survey to their respective veterinary mailing lists. This data collection was further supplemented by direct contact with individual veterinary practitioners.

In Ireland, the survey was mainly distributed through two intermediaries: an animal health organisation and a governmental department; they distributed the survey to their mailing list of veterinarians. This data collection was further supplemented by a press release announcing the survey and dissemination of the survey link through mainstream news articles and social media.

In Romania, the distribution of the survey was performed in two ways: direct contact of veterinarians by using social media tools and indirect contact of veterinarians by using the mailing list of the national veterinary autonomous, non-governmental organisation that coordinates the national activity of continuous professional development of veterinarians in Romania.

In Sweden, the survey was mainly distributed through two intermediaries: the Swedish Veterinary Association (SVF), which is a trade union and the professional organisation for Swedish veterinarians, and the District Veterinary Organisation (Distriktsveterinärerna, DV), which is an animal health provider with ambulatory and stationary practices all over Sweden and that is part of the Swedish Board of Agriculture. SVF distributed the survey to their mailing list of all members, and DV posted information on their intranet. This data collection was further supplemented by posts in social media groups for Swedish veterinarians.

The survey was hosted online via a commercial survey tool (SurveyMonkey Inc, San Mateo, USA). All data was collected anonymously, with no collection of any personalised or identifying data. Upon entering the survey platform, respondents were presented with information on the survey and asked to provide informed consent if they were willing to participate. Following, they completed a number of socio-demographic questions, including age, gender, level of education, area of residence, country of residence, number of years working as a vet, and the type of production system they worked with. Participants then completed a number of survey items measuring various psychological constructs (reported elsewhere: see Sweeney et al.) before completing a series of survey items measuring three facets of risk perception (perceived disease likelihood, perceived disease impact, and perceived disease control) for 59 sector-specific animal health diseases (16 diseases for cattle, 13 diseases for small ruminants, 16 diseases for pigs, and 14 diseases for poultry). The items used to measure the three facets of risk perception were adapted from previous survey studies [35, 48, 49]:

1. Perceived Likelihood of Disease: how likely they believed the disease would occur in their sector (very unlikely-very likely, 5-point scale);
2. Perceived Impact of Disease: how much of a negative impact they felt it would have if it did occur (very low impact-very high impact, 5-point scale);
3. Perceived Control of Disease: how confident they are it would be controlled if it did occur in their sector (it would be very easy-it would be very difficult, 5-point scale).

The disease lists were informed by previous work undertaken prioritising livestock diseases at the European level [45], and an extensive literature review on livestock diseases of importance at the European level [50] which was informed by an initial DISCONTOOLS (discontools.eu) disease prioritisation model (February 2023). If a veterinarian did not work in a particular sector, they could skip the risk perception questions for that sector and move to the next one.

Ethical approval for this study was obtained from the Social Research Ethics Committee (SREC) at Teagasc.

### Data cleaning and analysis

Data collection for the survey took place between May and November 2024, during which 845 vets started filling out the survey. During data cleaning, cases with incomplete responses or noticeable anomalies were addressed. Open-ended responses and comments were checked for suspicious answers, for example, selecting the same option for every single question in a section (termed “straight-lining”), and responses from respondents who gave an invalid date of birth were screened for irregularities. Survey duration was checked to identify respondents who completed too quickly. In total, 191 responses dropped out before or during the risk perception section, and these were removed from the dataset. One ‘test’ response from researchers, five responses from countries not included in the study sample, one duplicate respondent, and six straight-lined responses were also removed from the dataset after review on a case-by-case basis. After data cleaning, 624 respondents who completed at least the risk perception items were retained in the dataset. The survey was completed in full for at least one sector by 519 veterinarians, reflecting a completion rate of 61.42%, with 891 completed sector responses across respondents, accounting for those who completed more than one sector. In the disease prioritisation part of the survey, a response of ‘I am not aware of this disease’ within the risk perception part was re-coded to the same response to all three parameters (likelihood, impact and control of an individual disease) to reflect this lack of awareness and ensure consistency in responses to standardise for analysis. Statistical analysis was carried out in R (4.2.3) [51] and Microsoft Excel.

### Three-item disease risk perception score

To examine veterinarians’ perception of the risk of different diseases under consideration in this study, we made use of a three-item disease risk perception score. However, it is important to note that here we use the term ‘risk’ to denote this specific understanding of risk perception. This score combines three conceptually related but empirically distinct aspects of disease risk perception: the likelihood of an outbreak occurring, the perceived impact of an outbreak, and the perceived difficulty of controlling an outbreak. Initially, to calculate disease risk perception scores for specific diseases, responses were assigned integer values to the perceived likelihood of an outbreak occurring, its perceived impact, and the perceived difficulty of control as follows: very unlikely/very low impact/very easy to control was assigned 1, unlikely/low impact/easy to control was assigned 2, neutral/moderate impact/neither easy nor difficult was assigned 3, likely/high impact/hard to control was assigned 4, and very likely/very high impact/very hard to control was assigned 5. Calculation of perceived risk scores was carried out both for all respondents and for only those respondents who reported being aware of the disease. This leads to two scores, one for respondents aware of the disease and one for all respondents. Respondents who reported not being aware of the disease were assigned 0 if they were included in the analysis, which, when included, leads to an alteration of the score. Following this, an average risk score per question (likelihood, impact, control) was calculated by dividing the product of the number of participants who responded to each question by their respective response’s assigned integer [1–5] described above by the total number of respondents (including or not including those who reported not being aware of a given disease). This yielded a perceived risk score out of 5. We then added these scores together to yield an aggregated perceived risk score out of 15. This score was then transformed into a final aggregated risk score out of 100 by dividing the risk score obtained by 15 and multiplying by 100. Final aggregate risk scores were then used for downstream analysis and plotting in R (4.2.3) [51]. For individual, average, aggregate, and final risk scores, see Supplementary Table 1.

## Results

### Participation across Europe

**Fig. 1:**
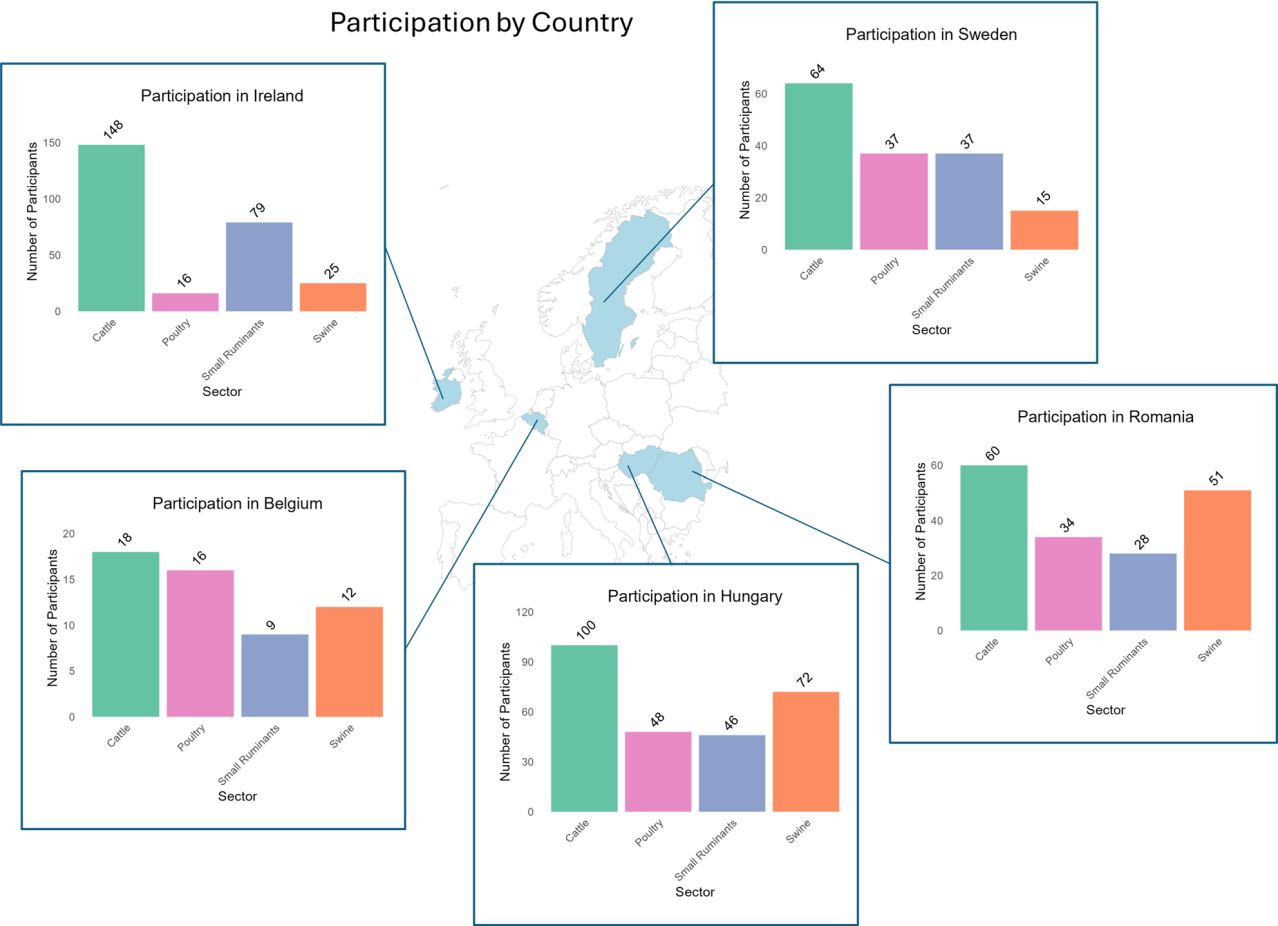
Participation in the survey by veterinarians in Belgium, Hungary, Ireland, Romania, and Sweden. Shaded countries indicate participation in the survey. Individual bar charts show the number of respondents who reported working in each of the following livestock production sectors: cattle, poultry, small ruminants, and pigs. The number of respondents is shown above the bars and indicates the number of respondents reporting to work within each sector; thus, the total number of respondents is not equal to the sum of responses per sector, as respondents may work in multiple sectors

A total of 624 responses were recorded from veterinarians participating in the survey, across five countries: Belgium, Ireland, Sweden, Hungary, and Romania (Fig. 1). Distribution was further attempted in Spain and Italy, although insufficient (*n* = < 5) responses across all sectors were recorded, and therefore, these were not included in analysis. The largest number of respondents were from the cattle sector in Ireland (*n* = 148), Hungary (*n* = 100), and Sweden (*n* = 64), and the small ruminant sector in Ireland (*n* = 79), Hungary (*n* = 46), and Sweden (*n* = 37). A low number of responses (*n* = < 20) was obtained from Belgium across all sectors.

### Veterinarian risk perception of diseases by sector and country

We initially examined the risk perception profiles of respondents based on their respective sectors and countries in which they reported working, taking into account the aggregated risk perception scores based on the respondents’ perception of the likelihood of an outbreak, the impact of the outbreak, and the difficulty of controlling an outbreak if it does occur. We further considered the overall risk score based on respondents’ perceptions depending on if they were aware or unaware of the disease in question. For a full breakdown of risk perception scores by country, see Supplementary Materials 1.

### Cattle and small ruminants

When carrying out the current survey and selecting the list of diseases to be included in the final questionnaire, we noted a high degree of overlap between the cattle and small ruminants’ sectors. Of the total number of veterinarians across all countries (*n* = 624), more than half (*n* = 397) reported working in the cattle sector, of which nearly a third (*n* = 121) also reported working in the small ruminant sector. With regards to the perceived risk for transboundary diseases, we observed a relatively high degree of agreement between the two sectors. This trend can be seen both at the national level, with bluetongue being perceived as the highest-risk disease for both cattle and small ruminants in Belgium, and at the European level, where FMD was seen as relatively high risk in Belgium, Romania, and Ireland. Conversely, veterinarians in both Sweden and Hungary perceived FMD pose a relatively similar level of risk to the sector as certain endemic diseases for both cattle and small ruminants. Data collection was carried out prior to the 2025 outbreak of FMD in Central Europe [8].

**Fig. 2:**
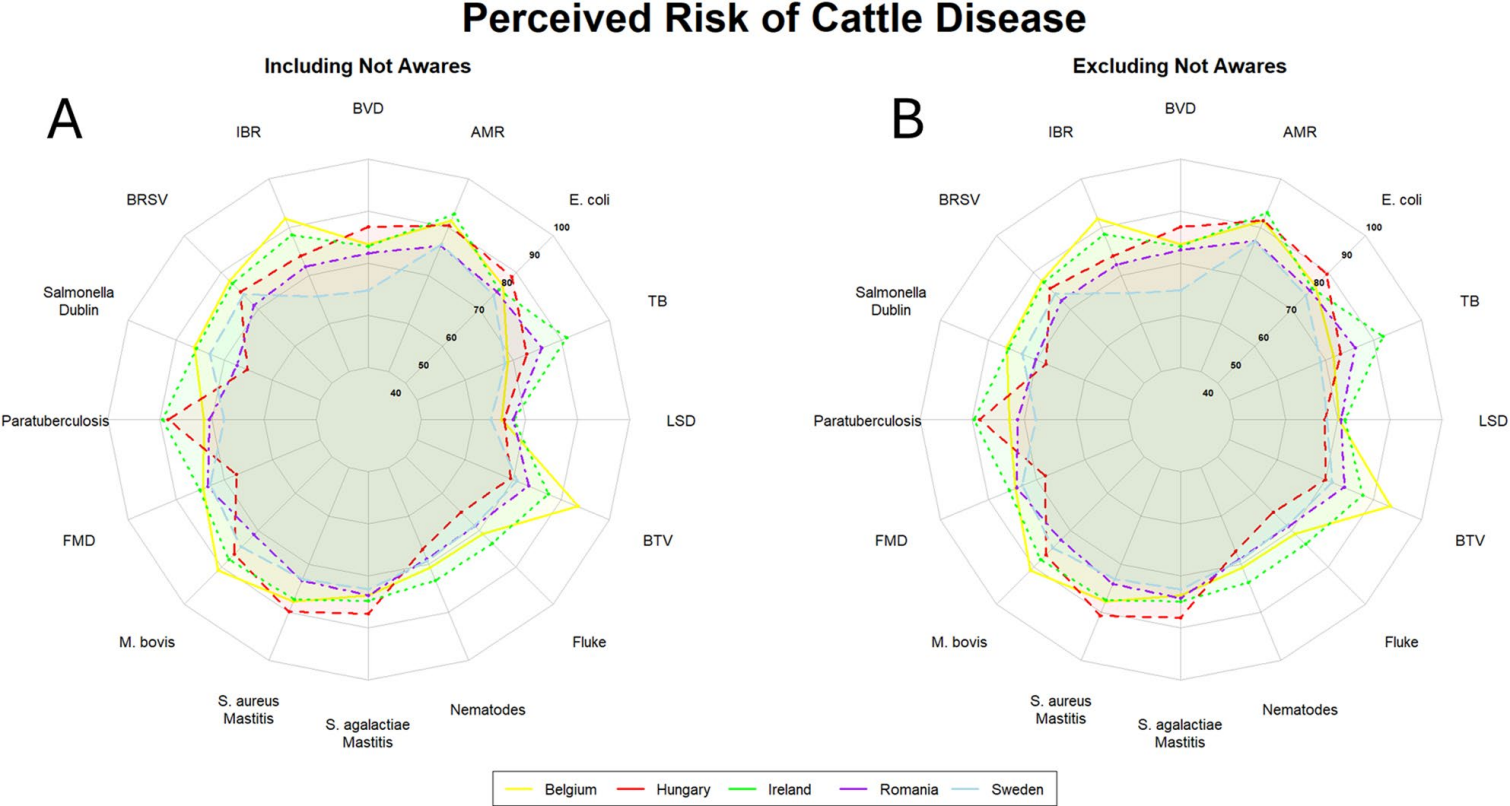
Radar plots showing the aggregated final risk perception scores per disease for veterinarians (Belgium [*n* = 18]; Hungary [*n* = 100]; Ireland [*n* = 148]; Romania [*n* = 60]; Sweden [*n* = 64]) working in the cattle sector. A: aggregated final risk scores for veterinarians working in the cattle sector. B: aggregated final risk scores for veterinarians working in the cattle sector, excluding those not aware of the disease in question. The number of respondents not aware of each specific disease is reported in Sect.  3.2. Countries are indicated by coloured lines and shaded areas. Belgium: yellow; Hungary: red; Ireland: green; Romania: purple; Sweden: light blue. Higher scores fall closer to the exterior edge and vice versa. List of abbreviations: BVD – bovine viral diarrhoea; AMR – antimicrobial resistance; TB – tuberculosis; LSD – lumpy skin disease; BTV – bluetongue virus; FMD – foot and mouth disease; BRSV – bovine respiratory syncytial virus

In the cattle sector specifically, bluetongue, infectious bovine rhinotracheitis (IBR), and AMR showed the highest levels of perceived risk in Belgium and Ireland, whereas in Romania and Hungary, bluetongue and IBR were not perceived to be high risk diseases (Fig. 2). Endemic diseases such as mastitis (*S. agalactiae* and *S. aureus*), and zoonotic diseases such as bovine tuberculosis, and *Salmonella* Dublin were also perceived as posing a high level of risk to the sector. The notable exception was Hungary, where *Salmonella* Dublin was perceived as posing a low risk to the sector. Lumpy skin disease was perceived as low risk by veterinarians in all countries examined, although as for FMD, data collection was completed prior to the further spread of LSD within Europe in 2025 [8]. The overall profile of perceived risk did not differ greatly when those who were not aware of the disease were excluded from the perceived risk score calculation.

When considering diseases affecting small ruminants, we also observed a high degree of variation. We observed a relatively even perception of the threat posed by endemic diseases or pathogens such as contagious pustular dermatitis (Orf), footrot, *E. coli*, *Coxiella burnetii*, and nematodes, considered to pose the highest risk in small ruminants. As for cattle, Belgian veterinarians perceived bluetongue as the highest risk disease for the sector. In general, transboundary diseases such as FMD, PPR, SGP, and bluetongue were not considered to pose a significant risk in the majority of countries surveyed. Romania was the only exception, where PPR was considered to pose the highest risk to the sector of any disease.

**Fig. 3:**
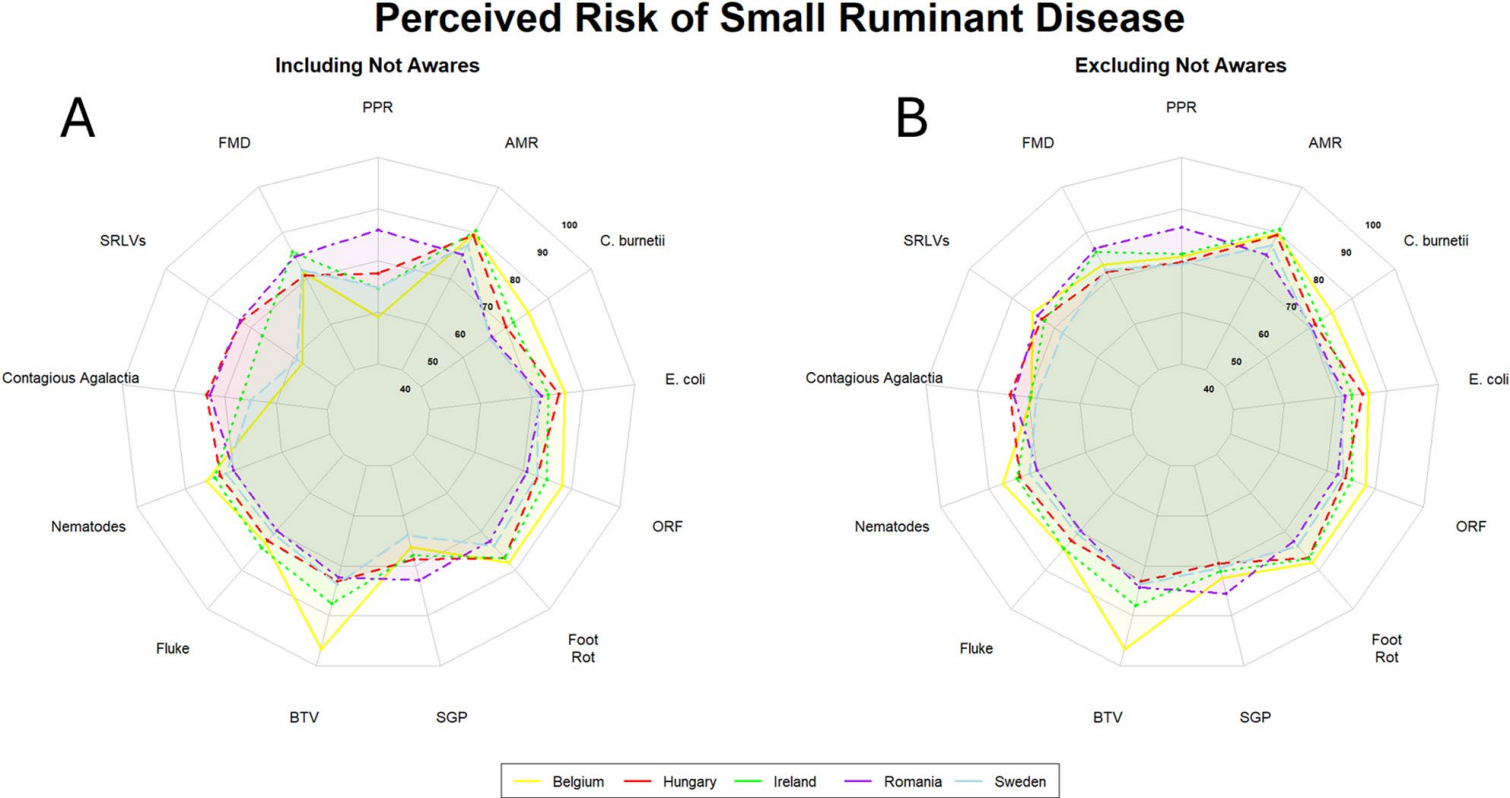
Radar plots showing the aggregated final risk scores per disease for veterinarians (Belgium [*n* = 9]; Hungary [*n* = 46]; Ireland [*n* = 79]; Romania [*n* = 28]; Sweden [*n* = 37]) working in the small ruminant sector. A: aggregated final risk scores for veterinarians working in the cattle sector. B: aggregated final risk scores for veterinarians working in the cattle sector, excluding those not aware of the disease in question. The number of respondents not aware of each specific disease is reported in Sect.  3.2. Countries are indicated by coloured lines and shaded areas. Belgium: yellow; Hungary: red; Ireland: green; Romania: purple; Sweden: light blue. Higher scores fall closer to the exterior edge and vice versa. List of abbreviations: PPR – peste des petits ruminants; AMR – antimicrobial resistance; SGP – sheep and goat pox; BTV – bluetongue virus; SRLVs – small ruminant lentiviruses; FMD – foot and mouth disease

We further observed a marked increase in the overall perceived risk scores for a number of small ruminant diseases when we removed the respondents who were not aware of the disease from the calculation of the aggregated risk scores, particularly for PPR, small ruminant lentivirus (SRLVs), and contagious agalactia (Fig. 3).

### Pigs

**Fig. 4:**
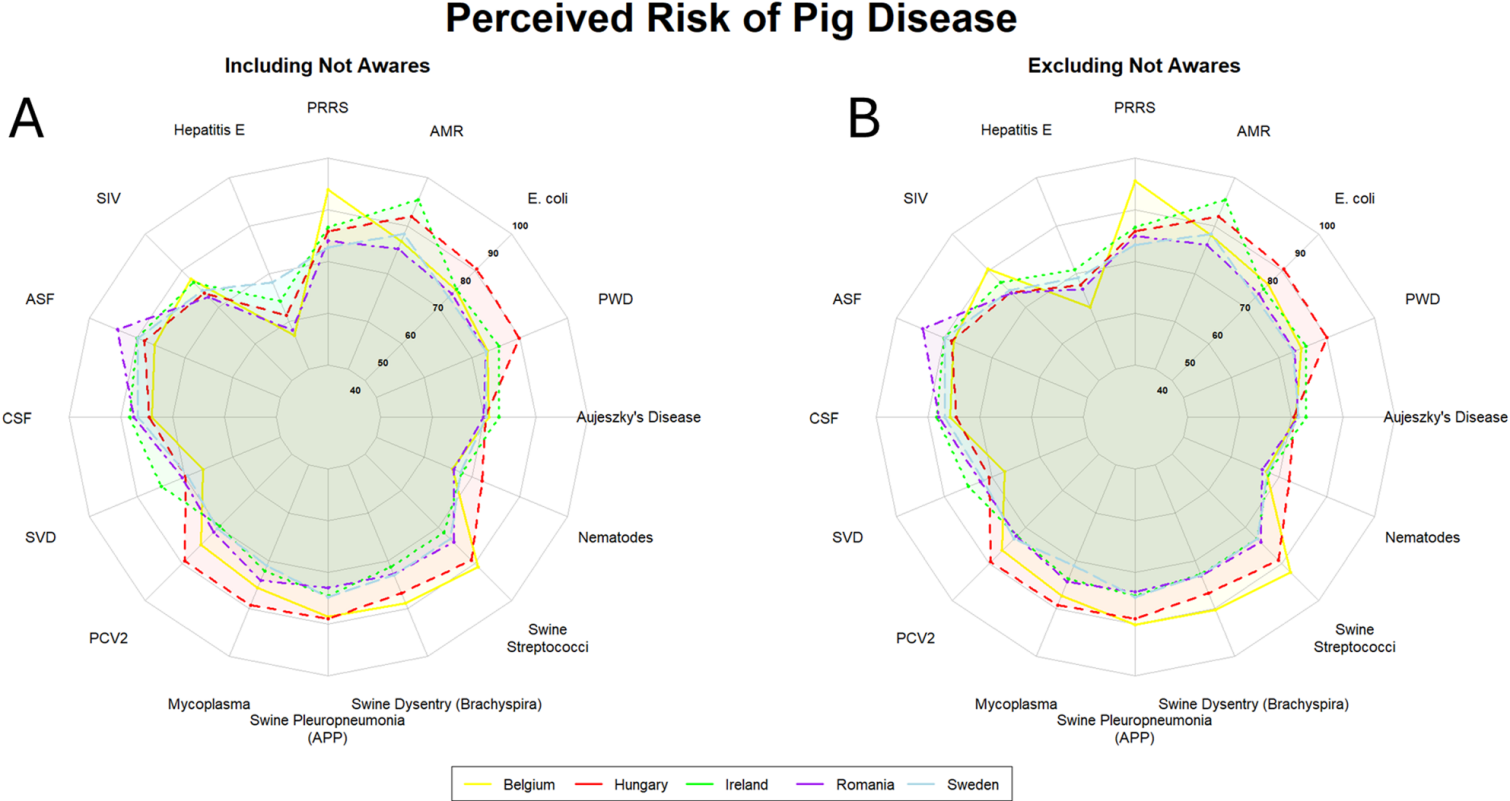
Radar plots showing the aggregated final risk scores per disease for veterinarians (Belgium [*n* = 12]; Hungary [*n* = 72]; Ireland [*n* = 25]; Romania [*n* = 51]; Sweden [*n* = 15]) working in the pig sector. A: aggregated final risk scores for veterinarians working in the pig sector. B: aggregated final risk scores for veterinarians working in the pig sector, excluding those not aware of the disease in question. The number of respondents not aware of each specific disease is reported in Sect.  3.2. Countries are indicated by coloured lines and shaded areas. Belgium: yellow; Hungary: red; Ireland: green; Romania: purple; Sweden: light blue. Higher scores fall closer to the exterior edge and vice versa. List of abbreviations: PRRS – porcine respiratory and reproductive syndrome; AMR – antimicrobial resistance; PWD – post-weaning diarrhoea; APP – *Actinobacillus pleuropneumoniae*; PCV2 – porcine circovirus type 2; SVD – swine vesicular disease; CSF – classical swine fever; ASF – African swine fever; SIV – swine influenza virus

Within the pig sector, we observed a country-specific profile for the diseases perceived as posing the highest risk, although overall the perception of a range of diseases was similar across all countries (Fig. 4). Nevertheless, although there was region- and country-specific variation in the perceived risk of a range of endemic diseases, ASF was perceived as one of the highest-risk diseases for the pig sector across all countries surveyed. It was only seen as the highest risk disease overall in Romania. In the remaining countries surveyed, endemic diseases were still seen as posing a higher risk. PRRS was seen as the highest risk disease in Belgium, and AMR in Ireland and Sweden. For Hungary and Belgium, a range of endemic bacterial infections, as well as AMR, were seen as posing a comparable risk to ASF. These included pig mycoplasmas (e.g., *Mycoplasma hyopneumoniae*), streptococci (e.g., *Streptococcus suis*), certain pathogenic strains of *E. coli* (e.g., Enterotoxigenic *E. coli*), and porcine pleuropneumonia (*Actinobacillus pleuropneumoniae*). Hepatitis E, swine vesicular disease (SVD), Aujeszky’s disease, and nematodes were seen as posing a low risk to the sector across all countries surveyed.

### Poultry

**Fig. 5:**
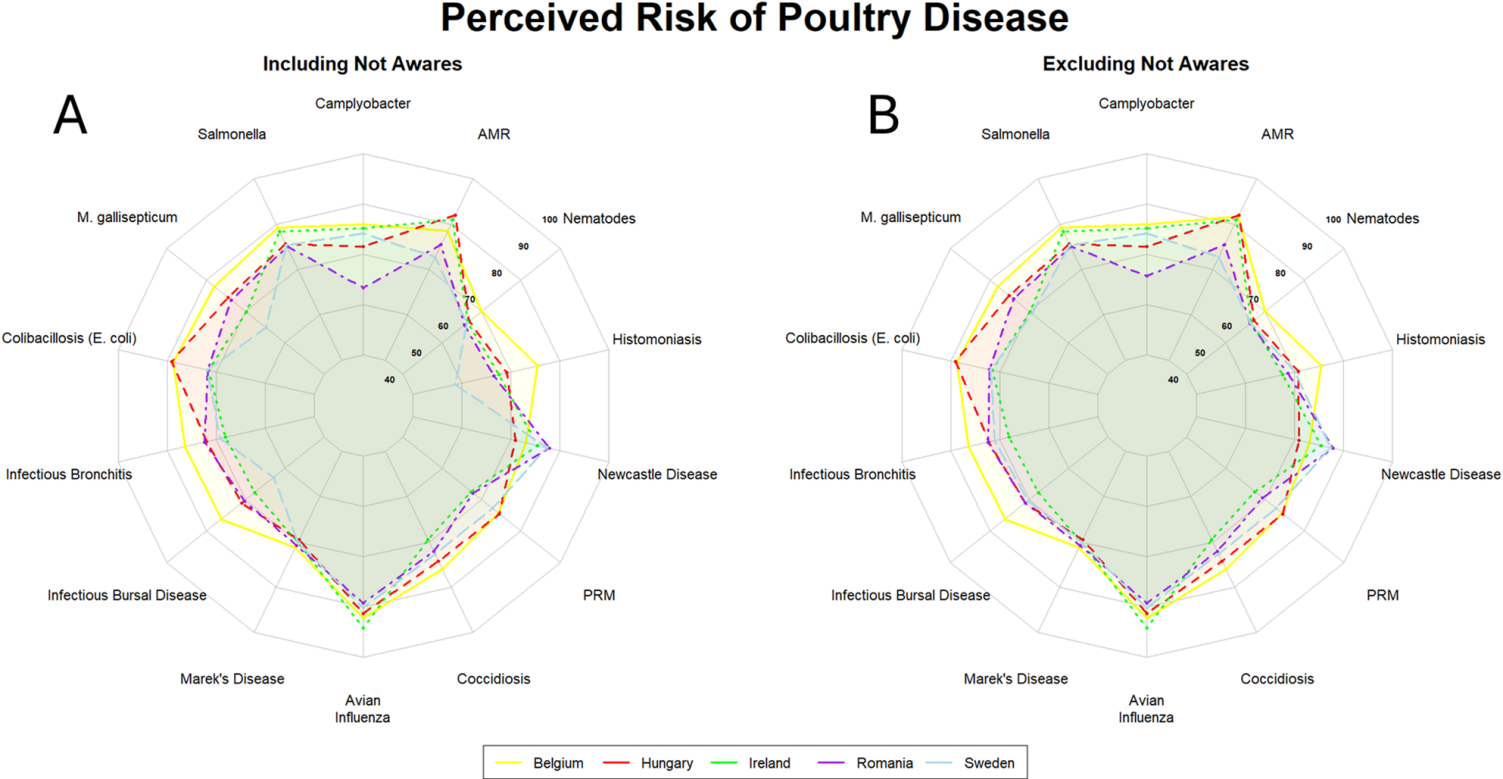
Radar plots showing the aggregated final risk scores per disease for veterinarians (Belgium [*n* = 16]; Hungary [*n* = 48]; Ireland [*n* = 16]; Romania [*n* = 34]; Sweden [*n* = 37]) working in the poultry sector. A: aggregated final risk scores for veterinarians working in the poultry sector. B: aggregated final risk scores for veterinarians working in the poultry sector, excluding those not aware of the disease in question. The number of respondents not aware of each specific disease is reported in Sect.  3.2. Countries are indicated by coloured lines and shaded areas. Belgium: yellow; Hungary: red; Ireland: green; Romania: purple; Sweden: light blue. Higher scores fall closer to the exterior edge and vice versa. List of abbreviations: AMR – antimicrobial resistance; PRM – poultry red mite

When considering poultry diseases, veterinarians across the countries surveyed in this study showed a broadly similar pattern of risk perception for the diseases included in the survey questionnaire (Fig. 5). Avian influenza, Newcastle disease, and AMR were perceived to pose the highest risk to the poultry sector overall. In both Hungary and Belgium, colibacillosis (*E. coli*) was perceived as one of the highest risk diseases for the poultry sector. Infectious bronchitis, histomoniasis, and infectious bursal disease were also seen as disease posing a high risk for the sector in Belgium. In contrast, histomoniasis was seen as posing a low risk to the poultry sector. Zoonotic bacterial infections, such as *Salmonella* and *Campylobacter*, were further perceived as posing a high risk to the sector in all countries, with the exception of *Campylobacter* in Romania, which was perceived as lower risk than all other countries. Nematodes were seen as posing a low risk to the poultry sector across all countries.

### Awareness gaps

**Fig. 6:**
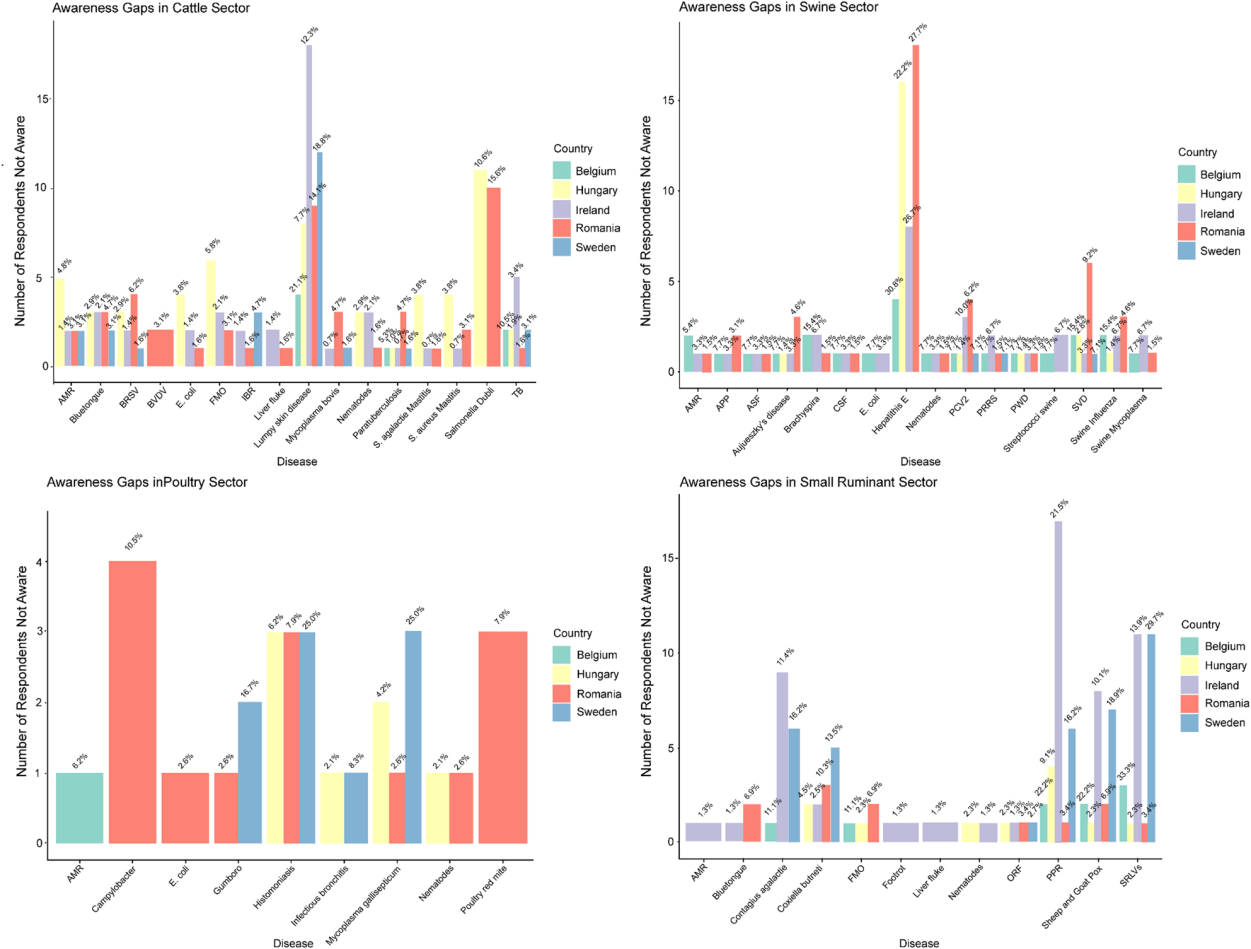
Bar charts showing awareness gaps for specific diseases by sector and by country (Belgium [teal]; Hungary [yellow]; Ireland [purple]; Romania [orange]; Sweden [blue]). Each individual bar chart represents a specific sector: **A**: cattle; **B**: pig; **C**: poultry; **D**: small ruminants. The x-axis indicates the disease for which an awareness gap was identified, and the y-axis indicates the total number of respondents per country who reported not being aware of the disease. The percentage of respondents who reported not being aware of the disease is indicated by the figures shown immediately above the country-specific bar. Only diseases for which an awareness gap was reported are shown. Bar width corresponds to disease and the number of countries where an awareness gap was reported. Wider bars indicate fewer countries showing an awareness gap, and vice versa

We then sought to examine the awareness gaps related to the diseases for which respondents were asked to rate their level of perceived risk of the disease to their sector (Fig. 6). Overall, we observed that the majority of included veterinarians reported being aware of most diseases in the questionnaire. However, there were a number of notable exceptions. In the cattle sector, the diseases for which there were the largest awareness gaps were lumpy skin disease, with respondents answering they were not aware of this disease in each country surveyed (7–18% of respondents, depending on the country). However, in general, we did not observe large awareness gaps for other transboundary diseases. We observed a relatively large awareness gap for *Salmonella* Dublin, with between 10 and 15% of respondents in Hungary, Ireland and Romania reporting they were not aware of this pathogen. A significant awareness gap for Hepatitis E was also observed for veterinarians in the pig sector, with between 20 and 30% of respondents in Belgium, Hungary, Romania, and Ireland reporting not being aware of this disease. Within the poultry and small ruminant sectors, we further observed awareness gaps of 10.5% for *Campylobacter* in Romania and 13.5% for *Coxiella burnetii* in Sweden respectively. We further observed a large awareness gap for small ruminant lentiviruses (SRLVs) in Sweden (29.7%). In the poultry sector, all Irish poultry veterinarians surveyed were aware of the diseases in the questionnaire. Belgian poultry veterinarians showed no awareness gaps other than for AMR, of which 6.2% of respondents reported not being aware. The greatest awareness gaps for poultry diseases were found in Sweden for *Mycoplasma gallisepticum*, histomoniasis (25% each), and infectious bursal disease (16.7%). We also observed awareness gaps in Romania for poultry red mite (7.9%).

## Discussion

In this study, we sought to carry out the first European level survey of veterinarian risk perception related to a range of infectious diseases considered to be of importance to the European livestock production sector. Overall, we received 624 complete responses from veterinarians in five (Belgium, Hungary, Sweden, Ireland, and Romania) countries. As expected, the number of responses varied by country and sector, as livestock production is not equally distributed across Europe. Generally, across all countries, we received the most responses from veterinarians working in the cattle sector, with all countries apart from Belgium showing response numbers of approximately 50–150 veterinarians. In the poultry and small ruminant sectors, conversely, we generally received lower response rates than for the cattle and pig sectors. These differences to some extent may be explained by the relative importance, size or numbers of veterinarians working in specific sectors within a given country. For the most part, this was in line with the dominance of particular sectors within each country. A notable exception was Romania, where responses from the small ruminant sector were disproportionately low. This may be due to the differing number of farms served by an individual veterinarian in the respective sectors or social factors of the animal owners of different animals making, e.g., small ruminant owners are less likely to have veterinary contact than pig and cattle owners.

In relation to the perception of risk for transboundary diseases, we note some results which merit consideration. In a prior study, it was noted that amongst respondents representing EU level stakeholder organisations, transboundary diseases were considered to pose the highest risk to all sectors [50]. In the current study, although the perception of the risk of FMD was high in Belgium, Romania, and Ireland, veterinarians in Sweden and Hungary considered it to pose a risk similar to that of many endemic diseases. In light of the recent outbreaks of FMD in Germany, Hungary and Slovakia [52, 53], which occurred after data collection (May – November 2024), it would thus be of value to consider this in follow-on work across Europe, and determine how perception of risk has changed following the recent central Europe outbreak. For PPR, which recently caused a devastating outbreak amongst small ruminants in Europe [52], our results are encouraging. Data collection for this study was completed prior to the spread of PPR to Greece, Romania, and subsequently Hungary via live animal transport from Romania in the summer of 2024 and early 2025 [8, 54]. Romanian veterinarians reported being aware of the risk posed by this disease. However, despite the recent outbreaks of SGP prior to the completion of data collection [54], awareness of SGP was limited, and there was a relatively low perception of the risk posed by the disease to the small ruminant sector. There was a high perceived risk of bluetongue in Belgium and Ireland. In Belgium, bluetongue was seen as the highest risk disease for both the cattle and small ruminant sectors. This can be attributed to the ongoing bluetongue outbreak in sheep and cattle which continuous to poses a threat to the cattle sector in Belgium, with large-scale vaccination campaigns currently ongoing. Hungary has previously successfully controlled bluetongue outbreaks in 2009 and 2014 with vaccination [55], which may explain the lower perception of risk for this disease. Romania self-declared freedom from Bluetongue virus (BTV) after implementing a comprehensive national monitoring and eradication programme [56]; this increased the Romanian veterinarians’ confidence in managing future outbreaks, and when cases were reported after September 2, 2020, the EU-mandated measures were successfully applied, with no further outbreaks reported through 2024 and into early 2025 [8, 54].

For pigs, ASF was widely seen as one of the diseases which posed the greatest risk to the sector. This was expected due to the devastating effect that ASF outbreaks have had on European pig production since the spread to the EU in the mid-2010s [57]. However, Romania was the only country where ASF was seen as the disease posing the greatest risk. Given the constant occurrence of ASF in Romania since 2017 [58–60], including spread to neighbouring countries [61, 62], this was to be expected. Thus, there may be a tendency amongst veterinarians working in EU frontier countries to perceive the risk of endemic diseases as lower than transboundary and epidemic diseases such as ASF, particularly due to the increased likelihood of spread from, and to, near neighbouring countries [63, 64]. The included Hungarian veterinarians also considered ASF to be high risk, which is in line with expectations due to the presence of the disease in wild boar in Hungary [65–67], and a border with Ukraine, Serbia, and Romania. Together, both CSF as ASF were seen by almost all veterinarians as posing a high risk to the sector, in line with the historic and ongoing outbreaks which have been caused by these diseases.

In poultry, avian influenza was seen as the disease posing the greatest risk to the sector across all countries examined. In light of ongoing outbreaks in Europe and their effects on the poultry industry [68–70], this was expected. Since 2020, Hungary has repeatedly been among the most affected of the five surveyed countries, experiencing particularly high numbers of HPAI outbreaks, while Belgium has also reported recurrent cases (69]. In the case of avian influenza, little variation was observed in the perceived risk between countries (Supplementary Materials 1), suggesting that there is broad agreement amongst the surveyed poultry veterinarians regarding the threat posed by avian influenza, and broad agreement between national level stakeholders and EU stakeholders [50, 71, 72]. In Hungary, the perceived risk is considered lower since vaccination of day-old chicks against Newcastle disease is compulsory [73]. In Sweden, in contrast, despite occasional outbreaks such as the commercial laying-hen case in 2023, disease freedom has been maintained through rapid eradication measures in the context of a non-vaccination national policy [74].

AMR was seen as posing a high risk to the livestock production sector as a whole by veterinarians in most countries. In Ireland, AMR was seen as one of the main sources of perceived risk which may be attributable to large-scale campaigns focusing on sustainable use of antimicrobials in the pig sector [75]. The Irish agricultural sector has been targeted strongly at the policy level in recent years [76, 77]. In Belgium, like Ireland, there have been concerted efforts to reduce AMR since 2012, with substantial reductions achieved [78]. Hungarian veterinarians also viewed AMR as posing a high risk to all sectors. This aligns with reports of increasing levels of resistance in Hungary [79], particularly in the poultry sector [80–82]; suggesting awareness of the risks of AMR is high within the country. The only exceptions to this general trend were in the pig and poultry sectors in Sweden and Romania. Although there are increasing reports of AMR, including multi-drug resistance, in Romania across many sectors [83–86], this may rather represent an aspect of the interpretation of the question, as AMR may not be seen as posing a significant risk to the health of the animals. Veterinarians in Sweden perceived AMR as a lower risk than in the remaining countries surveyed. This may be due, in the case of Sweden, to the relatively low incidence of AMR when compared to other European countries [87].

Contrary to EU level stakeholders [50], veterinarians overall considered endemic diseases to pose an important threat to their respective country’s sectors. Transboundary and epidemic diseases tend to occur in explosive epidemics leading to widespread, but relatively short lived, destruction, and are often subject to large scale control measures such as culling. On a daily basis, veterinarians are more likely to see the impact of endemic diseases. At the national level differences in perceived risk may be due to differing management systems. For IBR, we noted that Belgian veterinarians considered this disease to pose a high risk, whereas Romanian veterinarians saw IBR as posing a relatively low risk. This may, in part, reflect higher intensity management systems used in Belgium, compared to Romania. Increased density of cattle and longer periods of housing have been associated with a higher risk of the development of IBR [88–91]. Belgium initially aimed to be IBR-free by 2027 and planned to end vaccination by April 2025; however, due to a large increase in cases in 2024, this timeline was altered to 2030 for disease-free, and vaccination was allowed until 2027 [92]. We observed a similar variation in the perception of the risk posed by PRRS, which was seen as posing a high risk in Belgium, but relatively low in Romania. These results do not appear to correlate with prevalence, which is estimated at 20–25% in both Romania and Belgium [93, 94]. Conversely, in Hungary, despite a national eradication programme, achieving disease-free status in 2022 [95–97], there was still a relatively high perception of risk for PRRS. Sweden has been PRRS free since 2007 [98]. However, Swedish veterinarians reported a lower perception of risk for PRRS than Hungary. This suggests that the presence of a national eradication programme may lead to increased perception of disease risk, independent local prevalence, which may decrease as country maintains disease-free status.

Certain zoonotic diseases were also perceived as low risk. It is important, however, to note that we did not ask respondents to consider zoonotic risk. For example, Hepatitis E, although relatively prevalent in across Europe [99] is largely subclinical in pigs [100]. Workers in the pig sector do have a higher risk of exposure [101], and infection is a growing problem in Southeastern Europe [102]. However, Hepatitis E does not lead to mortality in most cases, except for certain vulnerable groups [102]. It is, nonetheless, of value in follow up work to consider the perceived zoonotic risk of livestock diseases. We noted an awareness gap of 27.7% (*n* = 14) for hepatitis E in Romania (*n* = 51). Once again, though, this must be taken with the caveat that the question may have been misinterpreted, and the sample size is small, affecting the inference that can be made.

We finally observed that many veterinarians worked across multiple sectors, and we found that the more sectors a veterinarian worked in, the more likely they were to report awareness gaps (Supplementary Materials 1). Thus, some awareness gaps might be attributed to veterinarians carrying out supplementary work in sectors which they are not as familiar with. However, we also note that, as we engaged in convenience sampling, this could be an artefact of this, and the large number of cattle veterinarians may also skew this data. We did not examine how many farms within each sector veterinarians visited, nor did we ask what the veterinarian’s primary sector was. This should also be examined in future work.

### Limitations

Finally, we would like to highlight a number of limitations to the current study. First and foremost, we made use of convenience sampling, with varying methodologies employed across the different countries. Therefore, these data may not be representative of the sector as a whole. We further, would highlight the relatively small sample sizes across countries, and the lack of uniformity of sample sizes per sector, thus, in many cases certain sectors are over-represented compared to others. This is to some extent due to convenience sampling, and the dominance of certain sectors in specific countries. However, this is a limitation which should be addressed in future work. Finally, although we endeavoured to cover a representative range of countries, a number of large EU countries are not represented, particularly Spain, France, Poland, Germany, and Italy, due to a combination of lack of participation (Spain, Italy, France), and lack of local project partners to carry out translation and survey distribution (Poland, Germany), therefore, these countries should be included in future work. Finally, we recognise that certain questions may have led to confusion, particularly regarding AMR and its potential impact, and regarding disease awareness, therefore, these aspects should be refined in future work.

## Conclusions

Here we present the findings of the first European-level survey of veterinarian disease risk perception and attitudes to specific livestock disease and awareness gaps within the poultry, pig, cattle, and small ruminant sectors. We found notable variation by country and a low perception of the risk posed by certain transboundary diseases in some countries, in addition to a low perception of the risk posed by infection with Hepatitis E. Our results further identified a range of potentially important awareness gaps with regard to specific diseases, including potentially lethal zoonotic infections and transboundary livestock diseases. We further observed a correlation between the number of sectors in which a veterinarian reported working and the number of awareness gaps. These results, thus, have important implications for disease control efforts and for One Health.

## Supplementary Information

Supplementary Material 1.

Supplementary Material 2.

Supplementary Material 3.

## Data Availability

Anonymised data available on request.
