# Supplementary material for: Veterinarians’ perception of livestock infectious disease: results from a five country cross-European survey (2024)
Source: BMC Vet Res. 2026 Mar 28;22:195. doi: 10.1186/s12917-026-05410-1 (PMC13036942; doi:10.1186/s12917-026-05410-1)
Supplement: Supplementary file 2 — Supplementary Material 2. [file 12917_2026_5410_MOESM2_ESM.docx]

**Supplementary Materials 1**

For analysis of the association between awareness gaps and the country or sector worked, ANOVA was used to determine statistical significance using p<0.05 as the threshold. For the association between the number of sectors worked and the number of reported awareness gaps, we made use of a linear regression model with p<0.05 as the threshold for statistical significance. Finally, for determining the association between working in one sector or multiple (>1.5), we made use of a t-test with p<0.05 as the threshold for statistical significance.

We sought to examine if there was a relationship between awareness gaps and the sector(s) and country in which veterinarians worked. We found that the country in which the veterinarians worked was not associated with more or fewer awareness gaps (p = 0.623). However, we did find that there was an association between the sector in which veterinarians worked and awareness gaps (p = 0.037), with the pig and small ruminants’ sectors being associated with higher awareness gaps than the cattle and poultry sectors. We further observed that many respondents (43%, n = 269) reported working in two or more different sectors. We therefore examined if working in multiple sectors was associated with awareness gaps. We found a positive correlation between the number of sectors in which a veterinarian reported working and the number of awareness gaps (r = 0.087), and although the overall correlation was relatively weak, it was judged to be significant (p = 0.044). We finally examined the pairwise relationship between awareness gaps and working in a single or multiple (>1) sectors, which was also found to be significant (p = 0.005).


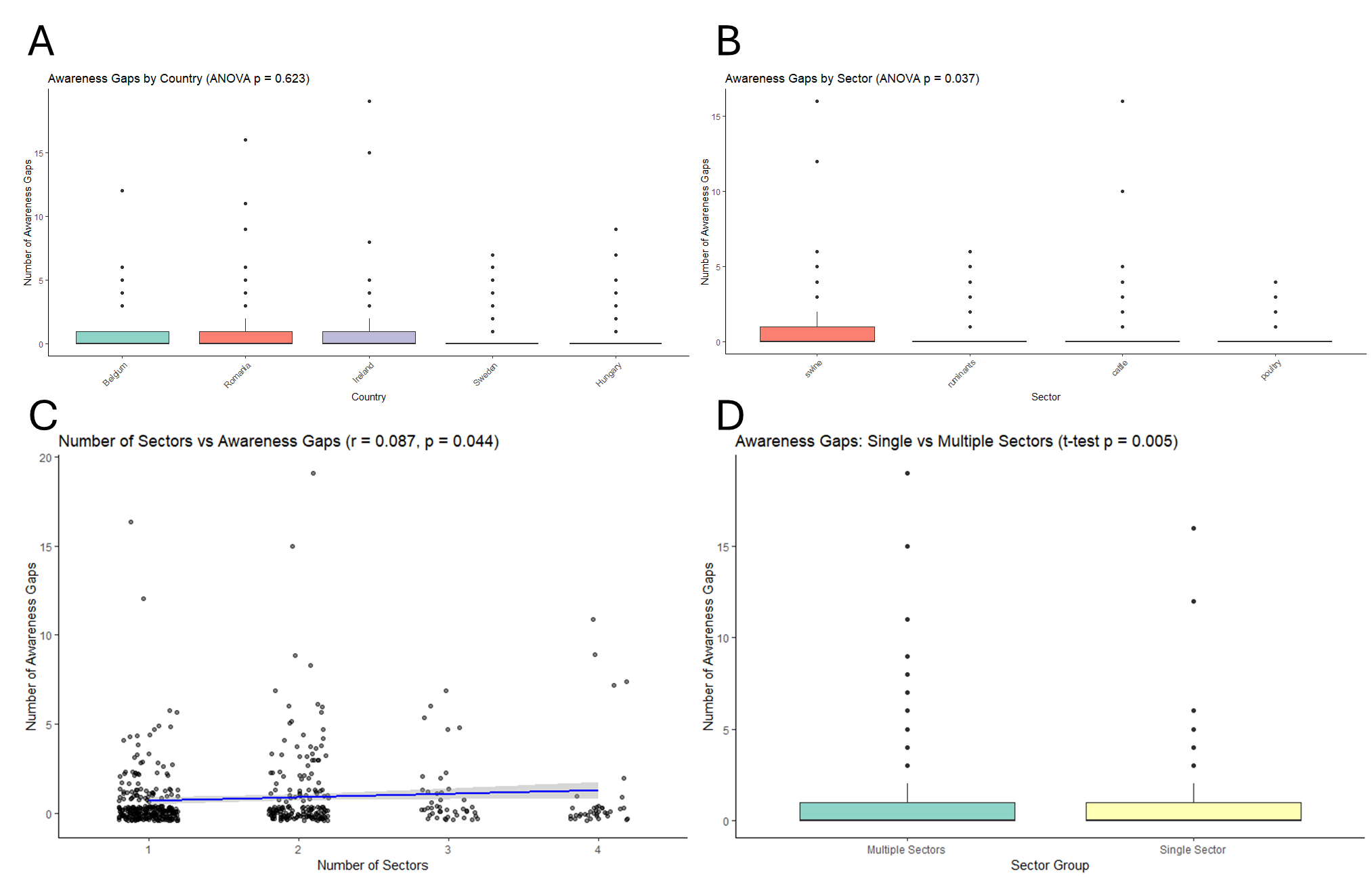


**Figure 7**: Association between awareness gaps and country and sector in which the veterinarian reported to work. A: box and whisker plot showing the number of awareness gaps (y-axis) by country (x-axis). B: box and whisker plots showing number of awareness gaps (y-axis) by sector (x-axis). C: linear regression model of the number of awareness gaps (y-axis) and the number of sectors in which the veterinarian reported working (x-axis). D: box and whisker plot showing the number of awareness gaps (y-axis) and whether the veterinarian reported working in a single sector or multiple sectors (x-axis).

To examine this further, we then examined the proportions of the total number of veterinarians working in the various sectors. Of those veterinarians who reported working in the pig sector (n = 194), approximately 36% (n = 70) worked exclusively in this sector, with a further 33% of respondents (n = 64) reporting working in three or more sectors. In the cattle sector (n = 397), a similar proportion (38%, n = 154) reported only working in this sector. Veterinarians who reported working in either the cattle or the cattle and small ruminant sectors alone constituted 66.4% (n = 264) of all respondents, with the remaining 33.5% (n = 133) working in three or more sectors. This is reinforced by the observation that of the veterinarians who reported working in the small ruminant sector (n = 198), the overwhelming majority (95%, n = 189) worked in two or more sectors, with less than 5% (n = 9) working exclusively in the small ruminant sector. However, this must be prefaced with the fact that 61% (n = 121) of veterinarians worked exclusively in the small ruminant and cattle sector, with a high degree of overlap of diseases between the two sectors. The remaining 34.3% (n = 68) worked in three or more sectors. Finally, among the total number of respondents who reported working in the poultry sector (n = 128), around 38% (n = 46) worked exclusively in the poultry sector, while a further 46% (n = 56) worked in three or more sectors.
