# Supplementary material for: Veterinarians’ perception of livestock infectious disease: results from a five country cross-European survey (2024)
Source: BMC Vet Res. 2026 Mar 28;22:195. doi: 10.1186/s12917-026-05410-1 (PMC13036942; doi:10.1186/s12917-026-05410-1)
Supplement: Supplementary file 3 — Supplementary Material 3. [file 12917_2026_5410_MOESM3_ESM.docx]

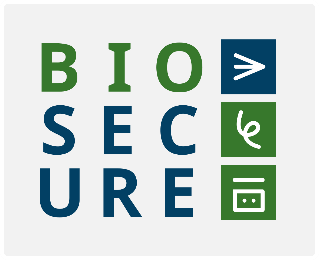


| **About the Survey** | | |
| --- | --- | --- |
| This survey aims to collect data for the Horizon Europe BioSecure project ([www.biosecure.eu](http://www.biosecure.eu)) which is funded by the Research Executive Agency of the European Commission. The overall goal of this project is to improve the capacity of people involved and working in livestock farming to understand, prioritise and implement evidence-based, cost-effective, and sustainable biosecurity management systems in the livestock production chain. The purpose of this questionnaire is to determine which diseases are most important to your local and national industry. The survey will help researchers to develop risk models and improved biosecurity knowledge for your industry.  We are reaching out to selected professionals whose expertise and experience are crucial in shaping a comprehensive understanding of biosecurity and disease prioritisation. Your unique perspective and insights are invaluable to us and will significantly contribute to the depth and quality of our findings. As such, your participation would not only enhance our study but also help in steering the conversation on this critical issue at a European level.  Participation in this study is completely voluntary and there can be no coercion in any way. You may refuse to participate in the study and you may withdraw from the study at any time without having to provide a reason. | | |
| **Data Management & Confidentiality** | | |
| Data storage and handling procedures are set in full compliance with the EU GDPR law (Regulation (EU) 2016/679). The data will be treated in a confidential manner and will exclusively be used for scientific purpose. During the course of the project the data collected will only be accessible by the research team. The data will be stored in a pseudonymised manner, meaning that your personal information (name, surname, address, email address, other) will be stored separately from the answers. Personal information collected in this survey will only be used to contact you again in case further information is needed. If the information obtained is published, it will be done in an anonymised way, this means no data will appear with which they can identify you. If you have more questions about this pseudonymised way of data collection and storage, please contact aine.regan@teagasc.ie. | | |
| **Consent Form** | | |
| **I consent to taking part in this survey:** | **Yes**  **No** |  |
| The research data collected here may also be useful in answering other research questions. Therefore, the possibility exists that the anonymous research data will be reused at a later date for other research. The reuse of the anonymous research data can be done both within the own research team, as well as by external researchers within and outside the European Union through a dedicated research data sharing platform. | | |
| **I consent to anonymised answers being transferred to other research projects:** | **Yes**  **No** |  |

| **1. Personal Information** | | | | | |
| --- | --- | --- | --- | --- | --- |
|  | | | | | |
| **Date of birth:** | ___/___/_______ |  | **Gender:** | Male / Female / other: | ____________ |
|  |  |  |  | |  |
| **Country of residence:** | ________________ |  | **Area of residence:** Urban / Rural / Suburban | | |
|  |  |  |  | |  |
| **Highest level of education:** | ________________ |  |  | |  |
|  |  |  |  | |  |

| **2. Which of the following most accurately describes your main role within agriculture:** (Please place a tick ✓) | |
| --- | --- |
| Farmer |  |
| Veterinarian |  |

| **3. Sector** (Please place a tick ✓) | | | | |
| --- | --- | --- | --- | --- |
|  | **Please select the animal production sector or sectors that are relevant to you** | | | |
|  | A. Cattle | B. Swine | C. Poultry | D. Small Ruminants (sheep, goats etc) |
|  |  |  |  |  |

| **4. Please rate the following** (Please place a tick ✓) | | | | | |
| --- | --- | --- | --- | --- | --- |
|  | **How would you rate the risk of infectious animal diseases on farms to the health of the farm animals?** | | | | |
|  | Very low risk | Low risk | Neutral | High risk | Very high risk |
|  |  |  |  |  |  |
|  | **How would you rate the risk of infectious animal diseases on farms to your own health?** | | | | |
|  | Very low risk | Low risk | Neutral | High risk | Very high risk |
|  |  |  |  |  |  |
|  | **How would you rate the risk of infectious animal diseases on farms to the health of the average person?** | | | | |
|  | Very low risk | Low risk | Neutral | High risk | Very high risk |
|  |  |  |  |  |  |
|  | **How would you rate the risk of infectious animal diseases on farms to the health of society?** | | | | |
|  | Very low risk | Low risk | Neutral | High risk | Very high risk |
|  |  |  |  |  |  |

| **5. Biosecurity is a set of practical measures to prevent the spread of disease on and between farms. How do you perceive each of the following statements?** (Please place a tick ✓) | | | | | |
| --- | --- | --- | --- | --- | --- |
|  | **I think biosecurity measures are worth implementing** | | | | |
|  | Strongly disagree | Disagree | Neither agree or disagree | Agree | Strongly agree |
|  |  |  |  |  |  |
|  | **Implementing biosecurity measures will improve the health of farm animals** | | | | |
|  | Strongly disagree | Disagree | Neither agree or disagree | Agree | Strongly agree |
|  |  |  |  |  |  |
|  | **Implementing biosecurity measures will improve the welfare of farm animals** | | | | |
|  | Strongly disagree | Disagree | Neither agree or disagree | Agree | Strongly agree |
|  |  |  |  |  |  |
|  | **Implementing biosecurity measures will improve the productivity of farm animals** | | | | |
|  | Strongly disagree | Disagree | Neither agree or disagree | Agree | Strongly agree |
|  |  |  |  |  |  |
|  | **Biosecurity needs to be improved in the farming industry as a whole** | | | | |
|  | Strongly disagree | Disagree | Neither agree or disagree | Agree | Strongly agree |
|  |  |  |  |  |  |

| **Cattle disease** | | | | | | |
| --- | --- | --- | --- | --- | --- | --- |
| **A6. How likely do you believe this disease may occur in your sector?** (Please place a tick ✓) | | | | | | |
|  | Very unlikely | Unlikely | Neutral | Likely | Very Likely | I am not aware of this disease |
| Bovine Viral Diarrhoea (BVD) |  |  |  |  |  |  |
|  |  |  |  |  |  |  |
| Infectious Bovine Rhinotracheitis (IBR) |  |  |  |  |  |  |
|  |  | | | | | |
| Bovine Respiratory Syncytial Virus (RSV) |  |  |  |  |  |  |
|  |  | | | | | |
| Salmonella Dublin |  |  |  |  |  |  |
|  |  | | | | | |
| Paratuberculosis (Johne’s disease) |  |  |  |  |  |  |
|  |  | | | | | |
| Foot and mouth disease |  |  |  |  |  |  |
|  |  | | | | | |
| Mycoplasma bovis |  |  |  |  |  |  |
|  |  | | | | | |
| *S. aureus* Mastitis |  |  |  |  |  |  |
|  |  | | | | | |
| *S. agalactiae* Mastitis |  |  |  |  |  |  |
|  |  | | | | | |
| Nematodes (Worms) |  |  |  |  |  |  |
|  |  | | | | | |
| Liver fluke |  |  |  |  |  |  |
|  |  | | | | | |
| Bluetongue |  |  |  |  |  |  |
|  |  | | | | | |
| Lumpy skin disease |  |  |  |  |  |  |
|  |  |  |  |  |  |  |
| Tuberculosis (TB) |  |  |  |  |  |  |
|  |  |  |  |  |  |  |
| E-Coli (Diarrhoea) |  |  |  |  |  |  |
|  |  | | | | | |
| Antimicrobial resistance (AMR) |  |  |  |  |  |  |
|  |  | | | | | |

| **A7. If this disease were to occur in your sector, how much of a negative impact do you think it would have?** (Please place a tick ✓) | | | | | | |
| --- | --- | --- | --- | --- | --- | --- |
|  | Very low impact | Low impact | Moderate impact | High impact | Very high impact | I am not aware of this disease |
| Bovine Viral Diarrhoea (BVD) |  |  |  |  |  |  |
|  |  |  |  |  |  |  |
| Infectious Bovine Rhinotracheitis (IBR) |  |  |  |  |  |  |
|  |  | | | | | |
| Bovine Respiratory Syncytial Virus (RSV) |  |  |  |  |  |  |
|  |  | | | | | |
| Salmonella Dublin |  |  |  |  |  |  |
|  |  | | | | | |
| Paratuberculosis (Johne’s disease) |  |  |  |  |  |  |
|  |  | | | | | |
| Foot and mouth disease |  |  |  |  |  |  |
|  |  | | | | | |
| Mycoplasma bovis |  |  |  |  |  |  |
|  |  | | | | | |
| *S. aureus* Mastitis |  |  |  |  |  |  |
|  |  | | | | | |
| *S. agalactiae* Mastitis |  |  |  |  |  |  |
|  |  | | | | | |
| Nematodes (Worms) |  |  |  |  |  |  |
|  |  | | | | | |
| Liver fluke |  |  |  |  |  |  |
|  |  | | | | | |
| Bluetongue |  |  |  |  |  |  |
|  |  | | | | | |
| Lumpy skin disease |  |  |  |  |  |  |
|  |  |  |  |  |  |  |
| Tuberculosis (TB) |  |  |  |  |  |  |
|  |  |  |  |  |  |  |
| E-Coli (Diarrhoea) |  |  |  |  |  |  |
|  |  | | | | | |
| Antimicrobial resistance (AMR) |  |  |  |  |  |  |
|  |  | | | | | |

| **A8. If this disease were to occur in your sector, how confident are you that it could be controlled?**  (Please place a tick ✓) | | | | | | |
| --- | --- | --- | --- | --- | --- | --- |
|  | It would be very easy | It would be easy | It would be neither easy nor difficult | It would be difficult | It would be very difficult | I am not aware of this disease |
| Bovine Viral Diarrhoea (BVD) |  |  |  |  |  |  |
|  |  |  |  |  |  |  |
| Infectious Bovine Rhinotracheitis (IBR) |  |  |  |  |  |  |
|  |  | | | | | |
| Bovine Respiratory Syncytial Virus (RSV) |  |  |  |  |  |  |
|  |  | | | | | |
| Salmonella Dublin |  |  |  |  |  |  |
|  |  | | | | | |
| Paratuberculosis (Johne’s disease) |  |  |  |  |  |  |
|  |  | | | | | |
| Foot and mouth disease |  |  |  |  |  |  |
|  |  | | | | | |
| Mycoplasma bovis |  |  |  |  |  |  |
|  |  | | | | | |
| *S. aureus* Mastitis |  |  |  |  |  |  |
|  |  | | | | | |
| *S. agalactiae* Mastitis |  |  |  |  |  |  |
|  |  | | | | | |
| Nematodes (Worms) |  |  |  |  |  |  |
|  |  | | | | | |
| Liver fluke |  |  |  |  |  |  |
|  |  | | | | | |
| Bluetongue |  |  |  |  |  |  |
|  |  | | | | | |
| Lumpy skin disease |  |  |  |  |  |  |
|  |  |  |  |  |  |  |
| Tuberculosis (TB) |  |  |  |  |  |  |
|  |  |  |  |  |  |  |
| E-Coli (Diarrhoea) |  |  |  |  |  |  |
|  |  | | | | | |
| Antimicrobial resistance (AMR) |  |  |  |  |  |  |
|  |  | | | | | |

| **Swine Disease** | | | | | | |
| --- | --- | --- | --- | --- | --- | --- |
| **B6. How likely do you believe this disease may occur in your sector?** (Please place a tick ✓) | | | | | | |
|  | Very unlikely | Unlikely | Neutral | Likely | Very Likely | I am not aware of this disease |
| Porcine respiratory and reproductive syndrome (PRRS) |  |  |  |  |  |  |
|  |  |  |  |  |  |  |
| Hepatitis E |  |  |  |  |  |  |
|  |  | | | | | |
| Swine Influenza |  |  |  |  |  |  |
|  |  | | | | | |
| African Swine Fever |  |  |  |  |  |  |
|  |  | | | | | |
| Classical swine fever |  |  |  |  |  |  |
|  |  | | | | | |
| Swine vesicular disease |  |  |  |  |  |  |
|  |  | | | | | |
| Porcine circovirus type II (PCV2) |  |  |  |  |  |  |
|  |  | | | | | |
| Mycoplasma |  |  |  |  |  |  |
|  |  | | | | | |
| Swine pleuropneumonia (APP) |  |  |  |  |  |  |
|  |  | | | | | |
| Swine Dysentery (Brachyspira) |  |  |  |  |  |  |
|  |  | | | | | |
| Swine Streptococci |  |  |  |  |  |  |
|  |  | | | | | |
| Nematodes (Worms) |  |  |  |  |  |  |
|  |  | | | | | |
| Aujeszky’s disease |  |  |  |  |  |  |
|  |  | | | | | |
| Post-weaning diarrhoea |  |  |  |  |  |  |
|  |  | | | | | |
| E-Coli |  |  |  |  |  |  |
|  |  | | | | | |
| Antimicrobial resistance (AMR) |  |  |  |  |  |  |
|  |  | | | | | |

| **B7. If this disease were to occur in your sector, how much of a negative impact do you think it would have?** (Please place a tick ✓) | | | | | | |
| --- | --- | --- | --- | --- | --- | --- |
|  | Very low impact | Low impact | Moderate impact | High impact | Very high impact | I am not aware of this disease |
| Porcine respiratory and reproductive syndrome (PRRS) |  |  |  |  |  |  |
|  |  |  |  |  |  |  |
| Hepatitis E |  |  |  |  |  |  |
|  |  | | | | | |
| Swine Influenza |  |  |  |  |  |  |
|  |  | | | | | |
| African Swine Fever |  |  |  |  |  |  |
|  |  | | | | | |
| Classical swine fever |  |  |  |  |  |  |
|  |  | | | | | |
| Swine vesicular disease |  |  |  |  |  |  |
|  |  | | | | | |
| Porcine circovirus type II (PCV2) |  |  |  |  |  |  |
|  |  | | | | | |
| Mycoplasma |  |  |  |  |  |  |
|  |  | | | | | |
| Swine pleuropneumonia (APP) |  |  |  |  |  |  |
|  |  | | | | | |
| Swine Dysentery (Brachyspira) |  |  |  |  |  |  |
|  |  | | | | | |
| Swine Streptococci |  |  |  |  |  |  |
|  |  | | | | | |
| Nematodes (Worms) |  |  |  |  |  |  |
|  |  | | | | | |
| Aujeszky’s disease |  |  |  |  |  |  |
|  |  | | | | | |
| Post-weaning diarrhoea |  |  |  |  |  |  |
|  |  | | | | | |
| E-Coli |  |  |  |  |  |  |
|  |  | | | | | |
| Antimicrobial resistance (AMR) |  |  |  |  |  |  |
|  |  | | | | | |

| **B8. If this disease were to occur in your sector, how confident are you that it could be controlled?**  (Please place a tick ✓) | | | | | | |
| --- | --- | --- | --- | --- | --- | --- |
|  | It would be very easy | It would be easy | It would be neither easy nor difficult | It would be difficult | It would be very difficult | I am not aware of this disease |
| Porcine respiratory and reproductive syndrome (PRRS) |  |  |  |  |  |  |
|  |  |  |  |  |  |  |
| Hepatitis E |  |  |  |  |  |  |
|  |  | | | | | |
| Swine Influenza |  |  |  |  |  |  |
|  |  | | | | | |
| African Swine Fever |  |  |  |  |  |  |
|  |  | | | | | |
| Classical swine fever |  |  |  |  |  |  |
|  |  | | | | | |
| Swine vesicular disease |  |  |  |  |  |  |
|  |  | | | | | |
| Porcine circovirus type II (PCV2) |  |  |  |  |  |  |
|  |  | | | | | |
| Mycoplasma |  |  |  |  |  |  |
|  |  | | | | | |
| Swine pleuropneumonia (APP) |  |  |  |  |  |  |
|  |  | | | | | |
| Swine Dysentery (Brachyspira) |  |  |  |  |  |  |
|  |  | | | | | |
| Swine Streptococci |  |  |  |  |  |  |
|  |  | | | | | |
| Nematodes (Worms) |  |  |  |  |  |  |
|  |  | | | | | |
| Aujeszky’s disease |  |  |  |  |  |  |
|  |  | | | | | |
| Post-weaning diarrhoea |  |  |  |  |  |  |
|  |  | | | | | |
| E-Coli |  |  |  |  |  |  |
|  |  | | | | | |
| Antimicrobial resistance (AMR) |  |  |  |  |  |  |
|  |  | | | | | |

| **Poultry Disease** | | | | | | |
| --- | --- | --- | --- | --- | --- | --- |
| **C6. How likely do you believe this disease may occur in your sector?** (Please place a tick ✓) | | | | | | |
|  | Very unlikely | Unlikely | Neutral | Likely | Very Likely | I am not aware of this disease |
| Campylobacter |  |  |  |  |  |  |
|  |  |  |  |  |  |  |
| Salmonella |  |  |  |  |  |  |
|  |  | | | | | |
| Mycoplasma gallisepticum |  |  |  |  |  |  |
|  |  | | | | | |
| Colibacillosis (E-Coli) |  |  |  |  |  |  |
|  |  | | | | | |
| Infectious bronchitis |  |  |  |  |  |  |
|  |  | | | | | |
| Infectious bursal disease (Gumboro) |  |  |  |  |  |  |
|  |  | | | | | |
| Marek’s disease |  |  |  |  |  |  |
|  |  | | | | | |
| Avian Influenza |  |  |  |  |  |  |
|  |  | | | | | |
| Coccidiosis |  |  |  |  |  |  |
|  |  | | | | | |
| Poultry red mite |  |  |  |  |  |  |
|  |  | | | | | |
| Newcastle disease |  |  |  |  |  |  |
|  |  | | | | | |
| Histomoniasis (Backhead) |  |  |  |  |  |  |
|  |  | | | | | |
| Nematodes (Worms) |  |  |  |  |  |  |
|  |  | | | | | |
| Antimicrobial resistance (AMR) |  |  |  |  |  |  |
|  |  | | | | | |

| **C7. If this disease were to occur in your sector, how much of a negative impact do you think it would have?** (Please place a tick ✓) | | | | | | |
| --- | --- | --- | --- | --- | --- | --- |
|  | Very low impact | Low impact | Moderate impact | High impact | Very high impact | I am not aware of this disease |
| Campylobacter |  |  |  |  |  |  |
|  |  |  |  |  |  |  |
| Salmonella |  |  |  |  |  |  |
|  |  | | | | | |
| Mycoplasma gallisepticum |  |  |  |  |  |  |
|  |  | | | | | |
| Colibacillosis (E-Coli) |  |  |  |  |  |  |
|  |  | | | | | |
| Infectious bronchitis |  |  |  |  |  |  |
|  |  | | | | | |
| Infectious bursal disease (Gumboro) |  |  |  |  |  |  |
|  |  | | | | | |
| Marek’s disease |  |  |  |  |  |  |
|  |  | | | | | |
| Avian Influenza |  |  |  |  |  |  |
|  |  | | | | | |
| Coccidiosis |  |  |  |  |  |  |
|  |  | | | | | |
| Poultry red mite |  |  |  |  |  |  |
|  |  | | | | | |
| Newcastle disease |  |  |  |  |  |  |
|  |  | | | | | |
| Histomoniasis (Blackhead) |  |  |  |  |  |  |
|  |  | | | | | |
| Nematodes (Worms) |  |  |  |  |  |  |
|  |  | | | | | |
| Antimicrobial resistance (AMR) |  |  |  |  |  |  |
|  |  | | | | | |

| **C8. If this disease were to occur in your sector, how confident are you that it could be controlled?**  (Please place a tick ✓) | | | | | | |
| --- | --- | --- | --- | --- | --- | --- |
|  | It would be very easy | It would be easy | It would be neither easy nor difficult | It would be difficult | It would be very difficult | I am not aware of this disease |
| Campylobacter |  |  |  |  |  |  |
|  |  |  |  |  |  |  |
| Salmonella |  |  |  |  |  |  |
|  |  | | | | | |
| Mycoplasma gallisepticum |  |  |  |  |  |  |
|  |  | | | | | |
| Colibacillosis (E-Coli) |  |  |  |  |  |  |
|  |  | | | | | |
| Infectious bronchitis |  |  |  |  |  |  |
|  |  | | | | | |
| Infectious bursal disease (Gumboro) |  |  |  |  |  |  |
|  |  | | | | | |
| Marek’s disease |  |  |  |  |  |  |
|  |  | | | | | |
| Avian Influenza |  |  |  |  |  |  |
|  |  | | | | | |
| Coccidiosis |  |  |  |  |  |  |
|  |  | | | | | |
| Poultry red mite |  |  |  |  |  |  |
|  |  | | | | | |
| Newcastle disease |  |  |  |  |  |  |
|  |  | | | | | |
| Histomoniasis (Blackhead) |  |  |  |  |  |  |
|  |  | | | | | |
| Nematodes (Worms) |  |  |  |  |  |  |
|  |  | | | | | |
| Antimicrobial resistance (AMR) |  |  |  |  |  |  |
|  |  |  |  |  |  |  |

| **Small Ruminant Disease** | | | | | | |
| --- | --- | --- | --- | --- | --- | --- |
| **D6. How likely do you believe this disease may occur in your sector?** (Please place a tick ✓) | | | | | | |
|  | Very unlikely | Unlikely | Neutral | Likely | Very Likely | I am not aware of this disease |
| Peste des Petits Ruminants |  |  |  |  |  |  |
|  |  |  |  |  |  |  |
| Foot and mouth disease |  |  |  |  |  |  |
|  |  | | | | | |
| Small ruminant lentiviruses |  |  |  |  |  |  |
|  |  | | | | | |
| Contagious agalactia |  |  |  |  |  |  |
|  |  | | | | | |
| Nematodes (worms) |  |  |  |  |  |  |
|  |  | | | | | |
| Liver fluke |  |  |  |  |  |  |
|  |  | | | | | |
| Bluetongue |  |  |  |  |  |  |
|  |  | | | | | |
| Sheep and goat pox virus |  |  |  |  |  |  |
|  |  | | | | | |
| Footrot |  |  |  |  |  |  |
|  |  | | | | | |
| Contagious Pustular Dermatitis (Orf) |  |  |  |  |  |  |
|  |  | | | | | |
| E-Coli |  |  |  |  |  |  |
|  |  | | | | | |
| Q fever (*Coxiella burnetii*) |  |  |  |  |  |  |
|  |  | | | | | |
| Antimicrobial resistance (AMR) |  |  |  |  |  |  |
|  |  | | | | | |

| **D7. If this disease were to occur in your sector, how much of a negative impact do you think it would have?** (Please place a tick ✓) | | | | | | |
| --- | --- | --- | --- | --- | --- | --- |
|  | Very low impact | Low impact | Moderate impact | High impact | Very high impact | I am not aware of this disease |
| Peste des Petits Ruminants |  |  |  |  |  |  |
|  |  |  |  |  |  |  |
| Foot and mouth disease |  |  |  |  |  |  |
|  |  | | | | | |
| Small ruminant lentiviruses |  |  |  |  |  |  |
|  |  | | | | | |
| Contagious agalactia |  |  |  |  |  |  |
|  |  | | | | | |
| Nematodes (Worms) |  |  |  |  |  |  |
|  |  | | | | | |
| Liver fluke |  |  |  |  |  |  |
|  |  | | | | | |
| Bluetongue |  |  |  |  |  |  |
|  |  | | | | | |
| Sheep and goat pox virus |  |  |  |  |  |  |
|  |  | | | | | |
| Footrot |  |  |  |  |  |  |
|  |  | | | | | |
| Contagious Pustular Dermatitis (Orf) |  |  |  |  |  |  |
|  |  | | | | | |
| E-Coli |  |  |  |  |  |  |
|  |  | | | | | |
| Q fever (*Coxiella burnetii*) |  |  |  |  |  |  |
|  |  | | | | | |
| Antimicrobial resistance (AMR) |  |  |  |  |  |  |
|  |  | | | | | |

| **D8. If this disease were to occur in your sector, how confident are you that it could be controlled?**  (Please place a tick ✓) | | | | | | |
| --- | --- | --- | --- | --- | --- | --- |
|  | It would be very easy | It would be easy | It would be neither easy nor difficult | It would be difficult | It would be very difficult | I am not aware of this disease |
| Peste des Petits Ruminants |  |  |  |  |  |  |
|  |  |  |  |  |  |  |
| Foot and mouth disease |  |  |  |  |  |  |
|  |  | | | | | |
| Small ruminant lentiviruses |  |  |  |  |  |  |
|  |  | | | | | |
| Contagious agalactia |  |  |  |  |  |  |
|  |  | | | | | |
| Nematodes (Worms) |  |  |  |  |  |  |
|  |  | | | | | |
| Liver fluke |  |  |  |  |  |  |
|  |  | | | | | |
| Bluetongue |  |  |  |  |  |  |
|  |  | | | | | |
| Sheep and goat pox virus |  |  |  |  |  |  |
|  |  | | | | | |
| Footrot |  |  |  |  |  |  |
|  |  | | | | | |
| Contagious Pustular Dermatitis (Orf) |  |  |  |  |  |  |
|  |  | | | | | |
| E-Coli |  |  |  |  |  |  |
|  |  | | | | | |
| Q fever (*Coxiella burnetii*) |  |  |  |  |  |  |
|  |  | | | | | |
| Antimicrobial resistance (AMR) |  |  |  |  |  |  |
|  |  | | | | | |

| **9. If there is a specific disease or pathogen that has not been included in the list, but you as a stakeholder feel is of critical importance to your sector please indicate this here:** | | | | |
| --- | --- | --- | --- | --- |
| _____________________________________ | | | | |
| ­­­ | | | | |
| **How likely do you believe this disease may occur in your sector?** (Please place a tick ✓) | | | | |
| Very unlikely | Unlikely | Neutral | Likely | Very Likely |
|  |  |  |  |  |
| { |  |  |  |  |
| **If this disease were to occur in your sector, how much of a negative impact do you think it would have?** (Please place a tick ✓) | | | | |
| Very low impact | Low impact | Moderate impact | High impact | Very high impact |
|  |  |  |  |  |
|  |  |  |  |  |
| **If this disease were to occur in your sector, how confident are you that it could be controlled?**  (Please place a tick ✓) | | | | |
| It would be very difficult | It would be difficult | It would be neither easy nor difficult | It would be easy | It would be very easy |
|  |  |  |  |  |
|  |  |  |  |  |

| **10. If you have any additional comments, you can include them here:** |
| --- |
|  |

**END**

**Thank you for your participation!**

**Your contribution is deeply valued and your insights will be essential to advancing our understanding in this critical field, helping us to prioritise disease and develop more effective strategies and policies around on-farm biosecurity. Should you wish to stay engaged with the outcomes of this study, the results will be published on our website https://biosecure.eu/results-and-outreach. Here you can subscribe to our newsletter and stay up-to-date with the latest BIOSECURE news.**
